# Supplementary material for: Iterative improvement in the automatic modular design of robot swarms
Source: PeerJ Comput Sci. 2020 Dec 7;6:e322. doi: 10.7717/peerj-cs.322 (PMC7924708; doi:10.7717/peerj-cs.322)
Supplement: Supplemental Information 3 [file peerj-cs-06-322-s003.zip › argos3/doc/api/standalone/a00595_source.html]

ARGoS: plugins/simulator/entities/wifi\_equipped\_entity.h Source File


- Main Page
- Related Pages
- Namespaces
- Classes
- Files

- File List
- File Members

# plugins/simulator/entities/wifi\_equipped\_entity.h

Go to the documentation of this file.

```
00001 
00007 #ifndef WiFi_EQUIPPED_ENTITY_H
00008 #define WiFi_EQUIPPED_ENTITY_H
00009 
00010 #include <argos3/core/simulator/entity/entity.h>
00011 #include <argos3/core/utility/math/vector3.h>
00012 
00013 namespace argos {
00014 
00015    class CWiFiEquippedEntity : public CEntity {
00016 
00017    public:
00018 
00019       ENABLE_VTABLE();
00020 
00021       CWiFiEquippedEntity(CComposableEntity* pc_parent);
00022 
00023       CWiFiEquippedEntity(CComposableEntity* pc_parent,
00024                           const std::string& str_id);
00025 
00026       virtual std::string GetTypeDescription() const {
00027          return "wifi";
00028       }
00029 
00030    };
00031 
00032 }
00033 
00034 #endif
```

---

Generated on 10 Jul 2018 for ARGoS by 
 1.6.1
